# From miscommunication to misinformation: *Streptomyces* species erroneously reported as producers of prodigiosin

**DOI:** 10.3389/fmicb.2025.1693024

**Published:** 2025-12-04

**Authors:** M. Kalim Akhtar

**Affiliations:** Department of Chemistry, College of Science, United Arab Emirates University, Al Ain, United Arab Emirates

**Keywords:** red pigment, bacteriology, secondary metabolite, prodiginine, illusory truth effect

Prodigiosin is a red-colored pigment produced in bacteria (Lu et al., 2024). This compound has attracted considerable interest due to its anticancer, antibiotic, antimalarial and even immunosuppressive activities (Darshan and Manonmani, 2015). On account of its promising biotechnological potential, the compound has been the focus of several reviews and experimental articles. The authors of these articles typically begin their discussion of prodigiosin by mentioning the names of a few bacterial species that are prominent producers of prodigiosin. In recent years, one name that has consistently cropped up as a prodigiosin-producer is *Streptomyces coelicolor*. This claim appeared in a highly cited 2021 review by Islan et al. (2022), repeated in a 2024 review by Lu et al. (2024) and, as recently as 2 months ago, reiterated by ul Huda et al. (2025), in what could be considered an authoritative review on prodigiosin. What all these reviews have in common is that their statement regarding *Streptomyces coelicolor* as a prodigiosin-producer is incorrect. This species is in fact a well-known producer of undecylprodigiosin (Tsao et al., 1985). Structurally, undecylprodigiosin differs from prodigiosin by the length of its alkyl side chain in which an undecyl group replaces the shorter pentyl chain found in prodigiosin.

So how did the false notion arise that *Streptomyces coelicolor* is a prodigiosin-producer? Well, it originated from the misleading title of a study carried out by Liu et al. (2017). The title of this paper is “*Metabolic engineering of Streptomyces coelicolor for enhanced prodigiosins (RED) production*.” In the title, the word “prodigiosin” is used interchangeably with the word “prodiginine” resulting in misinformation. The terms prodigiosin and prodiginine are not interchangeable, as they hold different meanings. Prodigiosin is the name of a chemically distinct molecule while prodiginine is the name given to the family of tripyrrole compounds, of which prodigiosin is a member. Hence, a more accurate title would have been, “Metabolic engineering of *Streptomyces coelicolor* for enhanced prodiginine (RED) production.”

*Streptomyces coelicolor* is not the only species to fall foul of this misattribution; *Streptomyces griseoviridis* offers us yet another example. As before, the error arises from the inaccurate title of an experimental study carried out by Kawasaki et al. (2008), “A prodigiosin from the roseophilin producer *Streptomyces griseoviridis*.” A thorough assessment of this work in addition to the follow-up study would have revealed that *Streptomyces griseoviridans* is a producer of prodigiosin R1 and prodigiosin R2 (Kawasaki et al., 2008; Kimata et al., 2018). These analogs are cyclic derivatives of prodigiosin in which the alkyl side chain is linked to the terminal pyrrole moiety. This structural arrangement differs markedly from the linear structure of the prodigiosin molecule.

However, inadvertent inaccuracies may also arise from misinterpretation of analytical data. The study by Ramesh et al. (2020) had initially revealed that *Streptomyces prasanthi*, previously known as *Streptomyces* sp. BSE6.1, was a prodigiosin-producer. However, subsequent genomic analysis identified it as a producer of undecylprodigiosin (Ramesh et al., 2021). Thus, performing whole-genome sequencing alongside comprehensive chemical profiling would reduce the likelihood of inaccurate conclusions.

In the world of psychology, such misattributions would be considered a classic case of the illusory truth effect, where repeated exposure of false information or misinformation eventually leads to its acceptance as fact (Fazio et al., 2015). Misinformation, if left unchallenged, can have serious consequences on shaping scientific ideas and even hinder scientific progress. An important lesson to be drawn here is that claims and statements, even seemingly simple one, need to be carefully evaluated before accepting them as fact. Doing so will help to ensure that scientific information is communicated with the highest rigor and accuracy.
